# Investigations to the Antibacterial Mechanism of Action of Kendomycin

**DOI:** 10.1371/journal.pone.0146165

**Published:** 2016-01-21

**Authors:** Yasser A. Elnakady, Indranil Chatterjee, Markus Bischoff, Manfred Rohde, Michaele Josten, Hans-Georg Sahl, Mathias Herrmann, Rolf Müller

**Affiliations:** 1 Department of Microbial Natural Products, Helmholtz Institute for Pharmaceutical Research Saarland, Helmholtz Centre for Infection Research and Department of Pharmaceutical Biotechnology, Saarland University, Saarbrücken, Germany; 2 Faculty of Science, Zoology Department, King Saud University, Riyadh, Saudi Arabia; 3 Institute of Medical Microbiology and Hygiene, Saarland University, Homburg, Germany; 4 Department of Medical Microbiology, Helmholtz Center for Infection Research, Braunschweig, Germany; 5 Department of Medical Microbiology, Bonn University, Bonn, Germany; Griffith University, AUSTRALIA

## Abstract

**Purpose:**

The emergence of bacteria that are resistant to many currently used drugs emphasizes the need to discover and develop new antibiotics that are effective against such multi-resistant strains. Kendomycin is a novel polyketide that has a unique quinone *methide ansa* structure and various biological properties. This compound exhibits strong antibacterial activity against Gram-negative and Gram-positive bacteria, including methicillin-resistant *Staphylococcus aureus* (MRSA). Despite the promise of kendomycinin in several therapeutic areas, its mode of action has yet to be identified.

**Methods:**

In this study, we used a multidisciplinary approach to gain insight into the antibacterial mechanism of this compound.

**Results:**

The antibacterial activity of kendomycin appears to be bacteriostatic rather than bactericidal. Kendomycin inhibited the growth of the MRSA strain COL at a low concentration (MIC of 5 μg/mL). Proteomic analysis and gene transcription profiling of kendomycin-treated cells indicated that this compound affected the regulation of numerous proteins and genes involved in central metabolic pathways, such as the tricarboxylic acid (TCA) cycle (SdhA) and gluconeogenesis (PckA and GapB), cell wall biosynthesis and cell division (FtsA, FtsZ, and MurAA), capsule production (Cap5A and Cap5C), bacterial programmed cell death (LrgA and CidA), the cellular stress response (ClpB, ClpC, ClpP, GroEL, DnaK, and GrpE), and oxidative stress (AhpC and KatA). Electron microscopy revealed that kendomycin strongly affected septum formation during cell division. Most kendomycin-treated cells displayed incomplete septa with abnormal morphology.

**Conclusions:**

Kendomycin might directly or indirectly affect the cell division machinery, protein stability, and programmed cell death in *S*. *aureus*. Additional studies are still needed to obtain deeper insight into the mode of action of kendomycin.

## Introduction

Natural products remain a primary source of therapeutic drugs [1–11] and are valuable tools for investigating and modulating cell biology. However, for these compounds to be effective tools, their modes of action and their molecular interactions with corresponding cellular target(s) must be understood. Kendomycin is a macrocyclic polyketide that is produced by several *Streptomyces* species, including *S*. *violaceoruber* [12]. In recent years, several reports have described methods for the synthesis of kendomycin, reflecting its significance in biological research [13–15]. Kendomycin exerts cytotoxic effects on a number of human tumor cell lines, including breast adenocarcinoma (MCF-7), stomach adenocarcinoma (HMO2), and hepatocellular carcinoma cell lines (HepG2). In addition, we previously reported that kendomycin interfered with mammalian proteasome activities and induced apoptosis in U937 cells [16]. Kendomycin displays strong antibacterial activity against Gram-negative and Gram-positive bacteria, and it is active against methicillin-resistant *Staphylococcus aureus* (MRSA) [17]. *S*. *aureus* is a common cause of hospital- and community-acquired infections [18–20]. This Gram-positive bacterium colonizes the anterior nares of at least one-third of the human population and causes a variety of infections ranging from simple wound infections to severe systematic infections, such as pneumonia and osteomyelitis. The pathogenicity of this organism is believed to depend largely on its ability to adapt to various host niches and to coordinate the expression of its large reservoir of virulence factors [21,22].

Owing to its potential as a lead compound in several therapeutic areas, we studied the molecular mechanism(s) of the antibacterial activity of kendomycin. To address this issue, we took a multidisciplinary approach, including radioactive labelling experiments, two-dimensional difference gel electrophoresis (2D-DIGE), gene expression profiling, and electron microscopy. Our results demonstrated that kendomycin inhibits the primary metabolic pathways of *S*. *aureus*, induces the formation of abnormal septa in dividing cells, and interferes with the expression of genes that encode proteins involved in programmed cell death.

## Results

### Determining the antibacterial activity of kendomycin

To determine the minimal inhibitory concentration (MIC) of kendomycin against the MRSA strain COL, which exhibits homogeneously high methicillin resistance[23], serial dilutions of kendomycin (diluted in methanol from 20 μg/mL to 0.156 pg/mL) were added to a constant number of COL cells (5 x 10^3^) in 96-well microplates, and the plates were incubated overnight at 37°C. These assays revealed that kendomycin had an MIC of 5 μg/mL against this MRSA isolate. An equivalent volume of methanol was used as a negative control.

**Fig 1:**
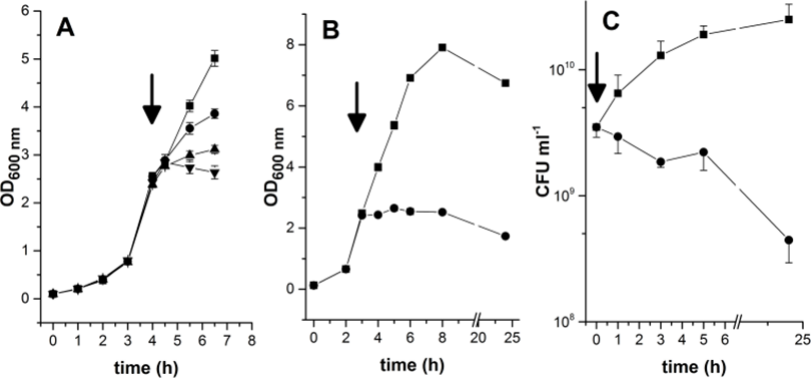
Effect of kendomycin on growth of *S*. *aureus* strain COL. Bacterial cultures were grown aerobically in BHI medium, and the optical densities at 600 nm of the cultures were measured at the time points indicated. Kendomycin addition is displayed by vertical arrows. Negative controls (■) were challenged with the solvent methanol at concentrations equivalent to the highest concentration added via the kendomycin supplementation. A) Effect of different kendomycin concentrations on growth of strain COL: 0.5 x MIC (●), 1x MIC (▲), and 3 x MIC (▼). The data presented are the mean ± SD of three independent experiments. B) Impact of kendomycin on the long-term growth characteristic of the *S*. *aureus* COL cell culture. BHI cultures of strain COL were grown for up to 24 h in absence (■) or presence of 3 x MIC kendomycin (●). The data presented are the mean ± SD of three independent experiments. C) Effect of kendomycin supplementation on the viability of the COL cell culture. Mid-exponential growth phase cells were either left untreated or challenged with 1.5 x MIC of kendomycin (●), and grown as outlined before. The number of viable bacteria were determined at the time points indicated by plating out serial dilutions of the cultures on agar plates. The data presented are the mean ± SD of two independent experiments.

Next, we studied the effects of different concentrations of kendomycin on the growth of the COL strain in liquid culture. We added a subinhibitory (0.5 x MIC) or inhibitory concentration (1 or 3 x MIC) of kendomycin to the COL cultures during the mid-exponential growth phase (OD_600_ = 2.5) (Fig 1A). We detected a concentration-dependent reduction in the growth yields of the cultures. The lowest examined concentration of kendomycin (0.5 x MIC) markedly decreased the OD_600_ compared to the control, and the highest concentration (3 x MIC) appeared to completely abolish the growth of these cultures; no increase in the OD_600_ was detected following the addition of kendomycin at this concentration (Fig 1B).

To evaluate the impact of kendomycin on cell viability, we next assayed the colony-forming units (CFU)/mL of COL cultures in the presence or absence of kendomycin. Unlike the control cultures, in which the CFU/mL consistently increased over time, steady decreases in the CFU/mL were detected in the drug-treated (1.5 x MIC) cultures (Fig 1C), which displayed a 56-fold decrease in viable cells after 24 h of growth compared with the control. However, there was a reduction in viable cells of less than one *log* after 24 h of incubation in 1.5 x MIC of kendomycin, suggesting a bacteriostatic rather than bactericidal activity of this compound against *S*. *aureus*.

### Effects of kendomycin on macromolecule synthesis

**Fig 2:**
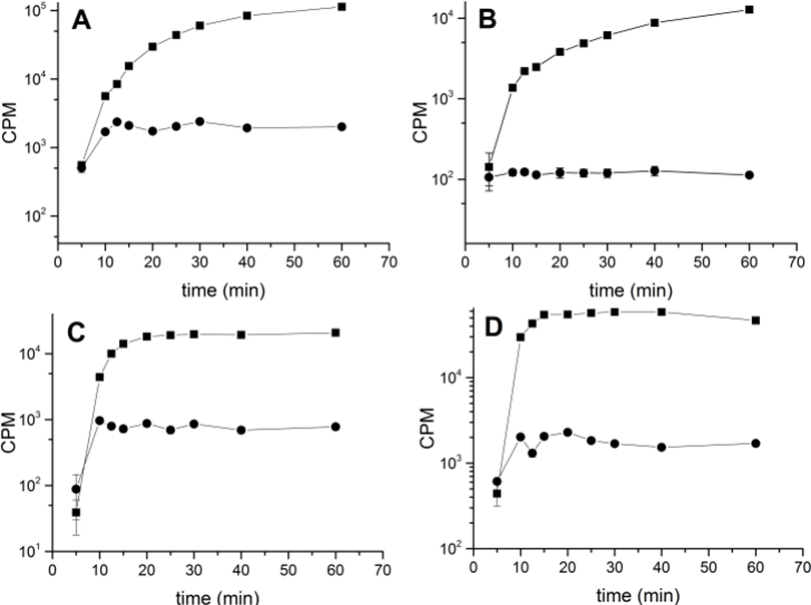
Effects of Kendomycin on the synthesis of macromolecules. The radioactive labeled precursors ^3^H-glucosamine (A), ^14^C-isoleucine (B), ^14^C-thymidin (C), and ^3^H-uridin (D) were added to an exponential growth phase culture of *S*. *aureus* strain COL in absence (■) and presence (●) of kendomycin (5 x MIC). Incorporation of labeled substances was determined as outlined in materials and methods. The data presented are the mean ± SD of two independent experiments.

We further studied the effects of kendomycin treatment on the biosynthesis of cellular macromolecules by the COL strain. The radiolabeled precursors ^3^H-glucosamine, ^14^C-isoleucine, ^3^H-uridine, and ^14^C-thymidine were fed to the growing COL cells to test the effects of kendomycin on cell wall, protein, RNA, and DNA synthesis, respectively. The incorporated radioactivity was then measured in the absence and presence of kendomycin (5 x MIC). The control cultures were treated with methanol. In contrast to the control cultures, in which all of the precursors were incorporated into their corresponding cellular macromolecules, kendomycin completely abolished the incorporation of all four radioactively labeled precursors (Fig 2). Similar results were obtained using the *Bacillus subtilis* 168 strain (data not shown). Antibiotics that target the bacterial energy transduction machinery to cause rapid energy depletion in cells typically simultaneously inhibit all biosynthetic pathways [24].

### Global proteome response to kendomycin treatment

To address whether kendomycin affects the protein expression pattern of the COL strain, two-dimensional (2D) gel electrophoresis followed by peptide mass fingerprinting was performed. Proteins were extracted from kendomycin-treated cells (1.5 x MIC) and control cells (treated with methanol) after 60 min of treatment. Each extracted protein sample was then labeled with one of three different fluorescent Cy dyes (Cy2, Cy3, and Cy5; Table 1) and was analyzed via fluorescent 2D-DIGE, as described in the Materials and Methods section. These experiments reproducibly identified approximately 2,000 spots corresponding to individual proteins. Ninety protein spots were affected by kendomycin treatment by a factor of ≥ 1.5-foldin all of the biological replicates (*P* < 0.001) and were excised from the gels. Of these 90 spots, 55 spots (61%) were up-regulated after kendomycin treatment; the other 35 spots (39%) were down-regulated. After the tryptic digestion of the excised proteins, the peptides were analyzed via matrix-assisted laser desorption/ionization-time of flight (MALDI-TOF) mass spectrometry, which identified only 61 spots. The identified spots included 24 spots corresponding to down-regulated proteins and 37 spots corresponding to up-regulated proteins (Table 2). However, many of the identified proteins were detected in more than one spot, which may have been due to either post-translational modifications or artificial modifications that occurred during the preparation of the protein samples.

**Table 1:** DIGE experimental design.

Gel-Nr.	Cy3	Cy5	Cy2
1	Untreated 1	Treated 3	Pooled STD*
2	Untreated2	Treated 4	Pooled STD*
3	Untreated 1	Treated 3	Pooled STD*
4	Untreated 2	Treated 4	Pooled STD*

*Pooled STD is an internal standard containing equal amounts of treated and untreated proteins from all samples in the experiment.

**Table 2:** Proteins that have been regulated after treatment of *S*. *aureus* COL cells with kendomycin.

Spot No.	Master no	Gene	Identified Protein	TheoreticalMWt	TheoreticalpI	Peptide count	Mascot score	Protein Score C.I. %	Average ratio
1	1264	*gapA2*	Glyceraldehyde3-phosphate dehydrogenase	37141	5.95	17	228	100	-2.6
2	779	*hutU*	Urocanatehydratase	60854	5.23	16	157	100	-2.54
3	1259	*gapA2*	Glyceraldehyde3-phosphate dehydrogenase	37141	5.95	11	160	100	-2.43
4	769	*hutU*	Urocanatehydratase	60854	5.23	10	82	97	-2.38
5	862	*pckA*	Phosphoenolpyruvatecarboxykinase	59511	5.74	22	234	100	-2.28
6	1263	*gapA2*	Glyceraldehyde3-phosphate dehydrogenase	37151	5.89	8	106	100	-2.27
7	849	*pckA*	Phosphoenolpyruvatecarboxykinase	59511	5.74	9	99	100	-2
8	851	*pckA*	Phosphoenolpyruvatecarboxykinase	59510	5.89	22	206	100	-1.91
9	1300	*gapA2*	Glyceraldehyde3-phosphate dehydrogenase	37151	5.89	16	194	100	-1.89
10	1257	*gapA2*	Glyceraldehyde3-phosphate dehydrogenase	37141	5.95	13	144	100	-1.83
11	1198	HP	Putative RNA methylasefamily (SACOL1483)	43621	5.07	12	139	100	-1.81
12	840	*fhs*	Formate-tetrahydrofolateligase	60017	5.76	11	145	100	-1.66
13	599	*thrS*	Threonyl-tRNAsynthetase	74455	5.26	23	189	100	-1.59
14	842	*fhs*	Formate-tetrahydrofolateligase	60017	5.76	16	192	100	-1.59
15	1068	*tuf*	Elongationfactor Tu	43134	4.74	22	327	100	-1.59
16	332	*kgd*	Alpha-ketoglutaratedecarboxylase	103104	5.47	15	135	100	-1.58
17	2109	*mecA*	Penicillin-bindingprotein 2'	24250	6.96	11	145	100	-1.58
18	589	*thrS*	Threonyl-tRNAsynthetase	74455	5.26	14	106	100	-1.57
19	930	*guaB*	Inosine-5'-monophosphate dehydrogenase	52904	5.61	9	90	99	-1.57
20	936	*guaB*	Inosine-5'-monophosphate dehydrogenase	52904	5.61	23	273	100	-1.52
21	766	*acsA*	Acetyl-CoAsynthetase	64593	5.1	12	119	100	-1.51
22	671	*pepF*	Oligoendopeptidase F	69890	5.14	19	187	100	-1.5
23	965	*glpK*	Glycerolkinase	55804	4.94	25	386	100	-1.5
24	1121	*tuf*	Elongationfactor Tu	43134	4.74	22	304	100	-1.5
25	850	* -*	1-pyrroline-5-carboxylate dehydrogenase	57003	4.98	26	405	100	1.5
26	507	*pflB*	Formateacetyltransferase	85264	5.31	21	249	100	1.54
27	1883	*- *	Acetoinreductase	27256	5.04	8	109	100	1.6
28	611	* - *	N-acetylmuramoyl-L-alanine amidase	69239	5.96	21	211	100	1.64
29	1274	* - *	ATP:guanidophosphotransferase	38699	5.09	9	120	100	1.64
30	506	*pflB*	Formateacetyltransferase	85264	5.31	22	243	100	1.71
31	865	*- *	Nicotinatephosphoribosyltransferase	54934	5.7	17	214	100	1.77
32	978	*- *	pyridinenucleotide-disulfideoxidoreductase	48500	5.54	11	127	100	1.86
33	733	*groEL*	ChaperoninGroEL	56263	4.61	14	184	100	1.89
34	861	*katA*	Catalase	58457	5.27	33	465	100	1.9
35	739	*groEL*	ChaperoninGroEL	57579	4.56	25	250	100	1.97
36	775	*sdhA*	Succinatedehydrogenaseflavoproteinsubunit	65633	5.4	21	196	100	1.97
37	847	*cscA*	Sucrose-6-phosphate hydrolase	58009	5.16	9	90	99	2
38	645	*dnaK*	DnaKprotein	66364	4.63	11	87	99	2.07
39	2132	HP	Putative nitroreductasedomain (SACOL2020)	23988	5.41	6	90	99	2.12
40	974	* - *	Pyridinenucleotide-disulfideoxidoreductase	48500	5.54	10	135	100	2.21
41	1364	HP	SACOL0409	39147	6.09	7	104	100	2.21
42	2094	*clpP*	ATP-dependentClpprotease	21557	5.13	8	86	99	2.23
43	1661	*grpE*	HeatshockproteinGrpE	23994	4.42	7	132	100	2.29
44	1630	*grpE*	HeatshockproteinGrpE	23994	4.42	8	115	100	2.45
45	750	*grpE*	HeatshockproteinGrpE	56263	4.61	23	284	100	2.49
46	740	*groEL*	ChaperoninGroEL	56263	4.61	16	180	100	2.58
47	647	*dnaK*	DnaKprotein	66364	4.63	25	320	100	2.96
48	447	*clpC*	Clp ATPase C	91069	5.51	16	195	100	4.01
49	663	*dnaK*	DnaKprotein	66364	4.63	20	288	100	4.19
50	449	*clpC*	Clp ATPase C	91069	5.51	36	393	100	5.04
51	997	* -*	Metallo-beta-lactamase family protein	49700	5.64	7	85	98	5.35
52	1997	*acpD*	Azoreductase	23350	4.95	11	142	100	5.38
53	2292	HP	Putative YceI-like domain protein	18643	4.74	12	184	100	5.93
54	441	*clpC*	Clp ATPase C	91069	5.51	19	172	100	5.99
55	452	*clpC*	Clp ATPase C	91069	5.51	39	398	100	6.17
56	1035	* -*	Metallo-beta-lactamase family protein	49551	5.61	7	99	100	6.34
57	499	*clpB*	ClpATPaseB	98357	4.97	24	193	100	6.53
58	536	*clpB*	ClpATPaseB	98357	4.97	19	171	100	6.59
59	357	*clpC*	Clp ATPase C	98385	4.97	34	318	100	6.88
60	353	*clpB*	Clp ATPase B	98357	4.97	17	121	100	7.55
61	500	*clpB*	Clp ATPase B	98357	4.97	32	299	100	10.29

The proteins that were affected by kendomycin treatment were involved in diverse cellular functions. The most strongly down-regulated proteins were involved in the tricarboxylic acid (TCA) cycle (PckA and SucA) [25], glycolysis/gluconeogenesis (GapB), amino acid metabolism (HutU and ThrS), nucleotide metabolism (GuaB), glycerolipid metabolism (GlpK), or protein synthesis (Tuf). These results suggest a general breakdown of the principal cellular metabolic pathways after treatment with kendomycin. Additionally, penicillin-binding protein 2 (PBP2) and MecA (syn. PBP2a) were down-regulated. The alternative penicillin-binding protein MecA is responsible for the MRSA phenotype and confers resistance to β-lactam antibiotics.

The most strongly up-regulated proteins were heat shock proteins (ClpB, ClpC, ClpP, GroEL, DnaK, and GrpE). The Clp ATPase ClpB was the most strongly up-regulated (more than 10-fold) protein in this category. The Clp complex is composed of a proteolytic subunit that associates with Clp ATPases. Whereas a Clp ATPase alone has a substrate-specific chaperone function (the refolding and reactivation of proteins), the Clp ATPase-ClpP complex confers protease activity. These enzymes are not only known to help bacterial cells compensate for various stress conditions but have also been suggested to have other physiological functions in *S*. *aureus* strains [26,27]. Other proteins that were up-regulated by kendomycin treatment included catalase (KatA), an enzyme that neutralizes H_2_O_2_ under oxidative stress conditions[28], and succinate dehydrogenase flavoprotein subunit A (SdhA), a component of the only enzyme complex of the TCA cycle that can directly feed electrons into the respiratory chain[29]. The up-regulation of SdhA after kendomycin treatment most likely occurred to compensate for the breakdown of energy metabolism.

### Gene expression profiling

We further investigated the effects of kendomycin treatment on the transcriptional expression patterns of selected genes in the COL strain. The aim of this experiment was to validate the data obtained from the proteomic analysis and to examine the expression levels of several other important genes that were not detected to be altered based on the proteomic analysis. The concentration of kendomycin, the incubation times, and all other experimental conditions were the same as those used in the proteomic analysis experiments.

**Fig 3:**
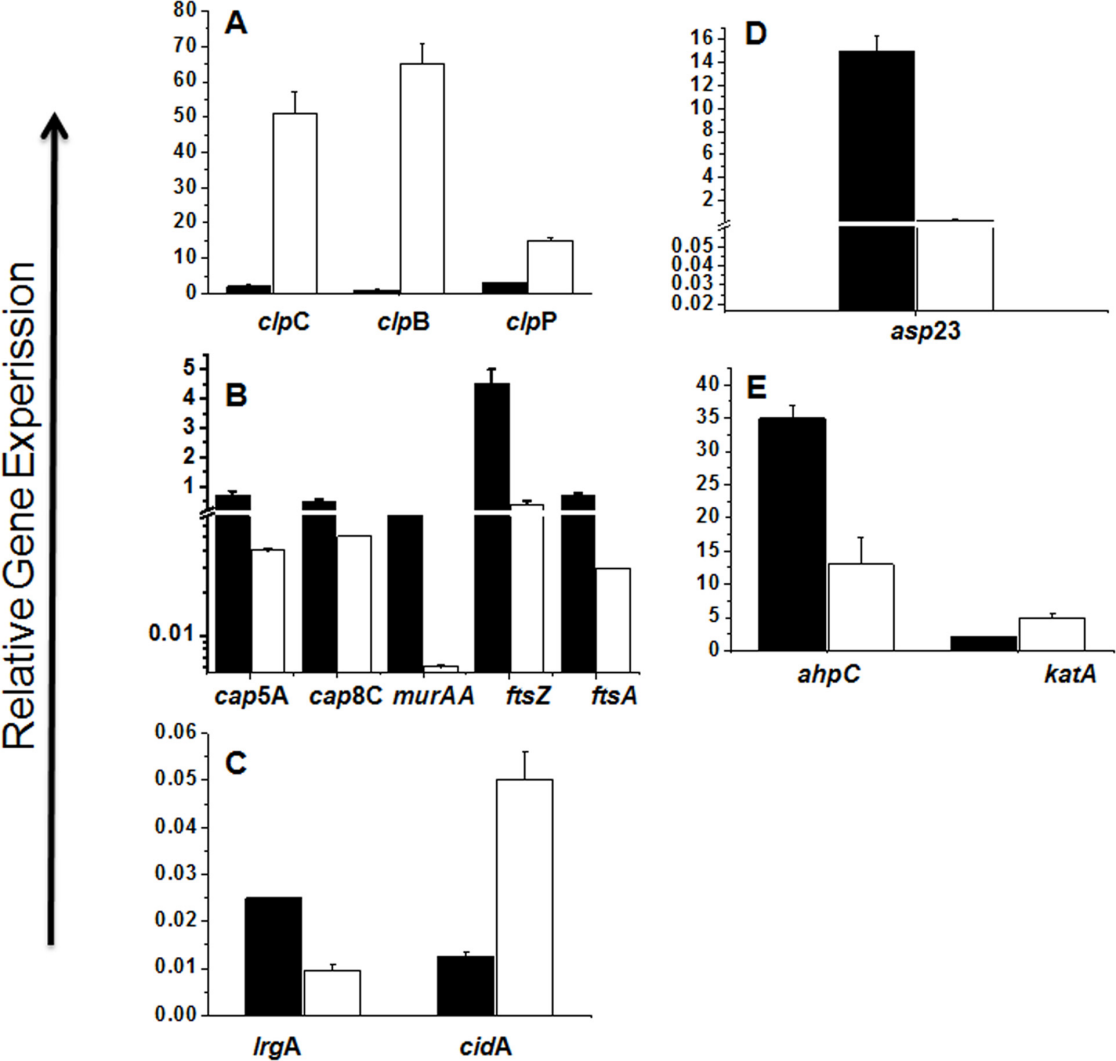
Effect of kendomycin on the transcription of selected genes in *S*. *aureus* COL. *S*. *aureus* COL cells were grown in BHI medium to mid-exponential growth phase, and subsequently supplemented with kendomycin (1.5 x MIC, white bars) or left untreated (black bars). One hour after drug addition, cells were harvested and used for total RNA isolation. A) Transcription of the heat shock protein encoding genes *clpC*, *clpB* and *clpP*. B) Transcription of the capsule operon genes *cap5A* and *cap8C*, the UDP-N-acetylglucosamine1-caboxyphenyltransferase encoding gene *murAA*, and the septum formation factors encoding genes *ftsA* and *ftsZ*. C) Transcription of the programmed cell death factor encoding genes *cidA* and *lrgA*. D) Transcription of the Sigma^B^ activity marker gene *asp23*. E) Transcription of the H_2_O_2_ inactivating factor encoding genes *ahpC* and *katA*. mRNA levels are expressed relative to gyrase B (in numbers of copies per copy of *gyrB*). The data presented are the mean ± SD of four independent experiments.

In accordance with the proteomic analysis, gene expression profiling revealed that molecular chaperones (*clpC*, *clpB*, and *clpP*) were the most strongly up-regulated genes after kendomycin treatment (Fig 3A). This finding might reflect an adaptation of the bacteria to compensate for stress conditions. In contrast, genes that are responsible for capsule production (*cap5A* and *cap5C*) [30] and cell wall biosynthesis (*murA*, *ftsA*, and *ftsZ*) were down-regulated in the kendomycin-treated cells (Fig 3B). FtsZ and FtsA are known to play critical roles in the early stages of bacterial cell division; they are among the first proteins to move to the division site [31–33].

In *S*. *aureus*, the *cidA* and *lrgA* genes have been shown to affect cell death during planktonic growth [34]. As shown in Fig 3C, the treatment of COL cells with kendomycin enhanced the expression level of *cidA* while reducing the expression level of *lrgA* (Fig 3C). Furthermore, we detected a decrease in the expression level of the alkaline shock protein 23 (*asp23*) gene. In *S*. *aureus*,*asp23* transcription is typically used as a marker for the activity of the alternative sigma factor Sigma^B^[35], which responds to several stress conditions by transiently increasing its activity [36,37], suggesting that kendomycin exerts a negative effect on Sigma^B^ activity. We further investigated the effect of kendomycin on the expression levels of two additional genes that encode proteins involved in the oxidative stress response in *S*. *aureus*, KatA and alkyl hydroperoxide reductase (AhpC) [38], which are regulated by the peroxide response regulator PerR [39]. As shown in Fig 3E, kendomycin induced the expression of *katA*, which supported the data obtained from the 2D-DIGE experiments. However, the expression level of *ahpC* was reduced after kendomycin treatment. AhpC is a member of the peroxiredoxin family, the members of which are known to detoxify H_2_O_2_, organic peroxides, and peroxynitrite. Both *ahpC* and *katA* exhibit compensatory regulation, with both independent and linked functions [38]. The opposing effects of kendomycin on *katA* and *ahpC* transcription suggest a PerR-independent mechanism.

### Effects of kendomycin on *S*. *aureus* cell morphology and ultrastructure

**Fig 4:**
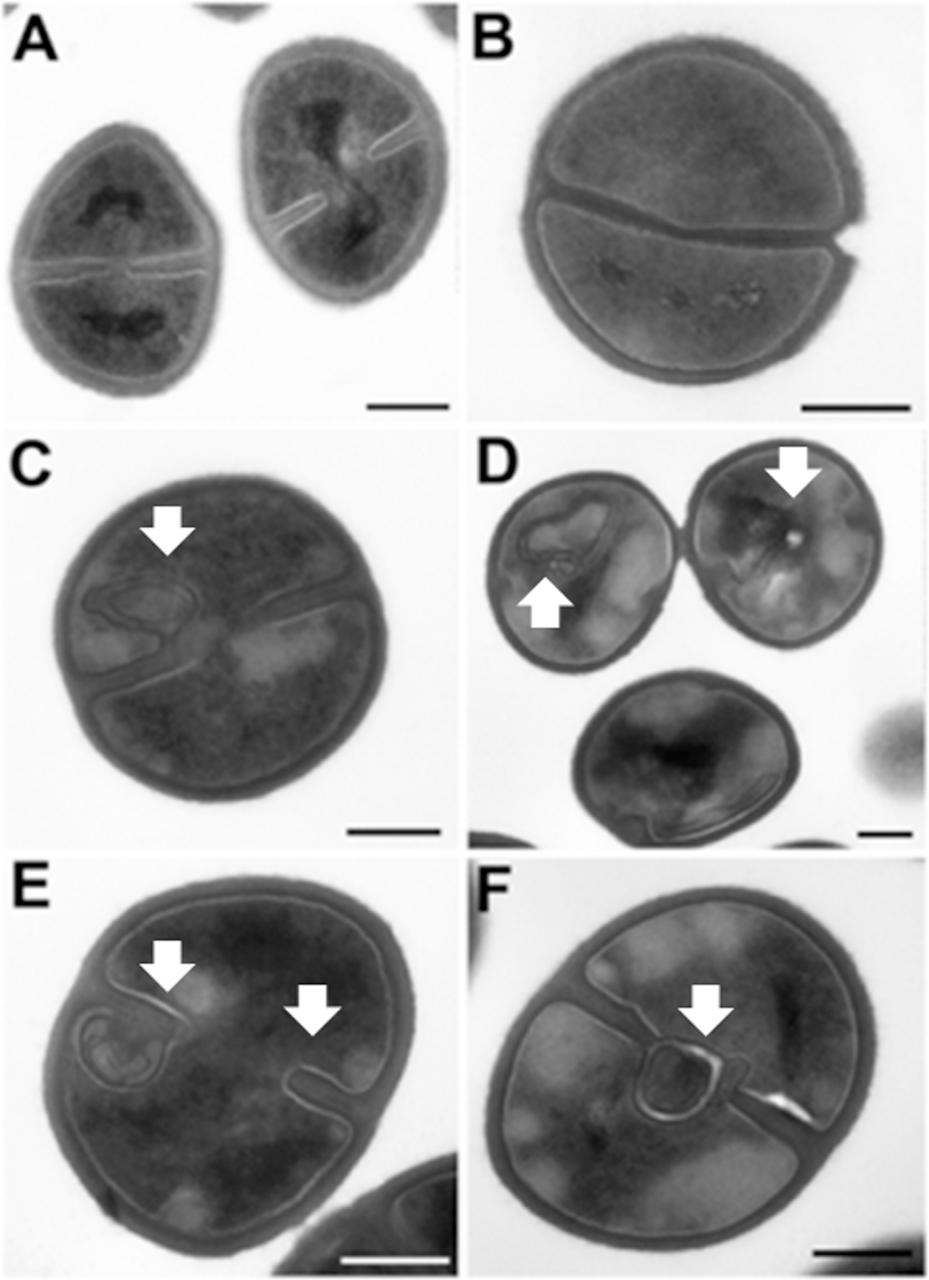
Effect of kendomycin on the cell division in *S*. *aureus* COL. Electron microscopic images of healthy control cells (A, B) and kendomycin treated cells (C-F). (A) Dividing control cells at the mid of septum formation. (B) Dividing cells with a completed septum. (C-F)Kendomycin treated cells exhibiting irregular septum morphologies. Bar = 200 nm.

The down-regulation of all of the examined genes involved in cell wall biosynthesis led us to suspect an effect of kendomycin treatment on the ultrastructure of *S*. *aureus* strain COL cells. In fact, transmission electron microscopic analysis of kendomycin-treated cells revealed alterations in the septation process in treated cells compared with the control cells (Fig 4), indicating direct or indirect inhibition of the cell division machinery. However, as illustrated in Fig 4C–4F, *S*. *aureus* COL cells remained intact after treatment with kendomycin, suggesting that the antibacterial mechanism of action does not involve cell lysis; this result was recapitulated via scanning electron microscopy (data not shown). The most characteristic morphological change induced by kendomycin treatment was the formation of irregular septa, suggesting that kendomyci ninhibits the cell septation system and induces the formation of fibrillar wall materials. Similar (but not identical) staphylococcal cell wall structures caused by the treatment of cells with various antibiotics, such as chloramphenicol [40], oritavancin [41] and penicillin, have been previously reported [42–44].

### Effect of kendomycin on *S*. *aureus* COL acetate consumption

**Fig 5:**
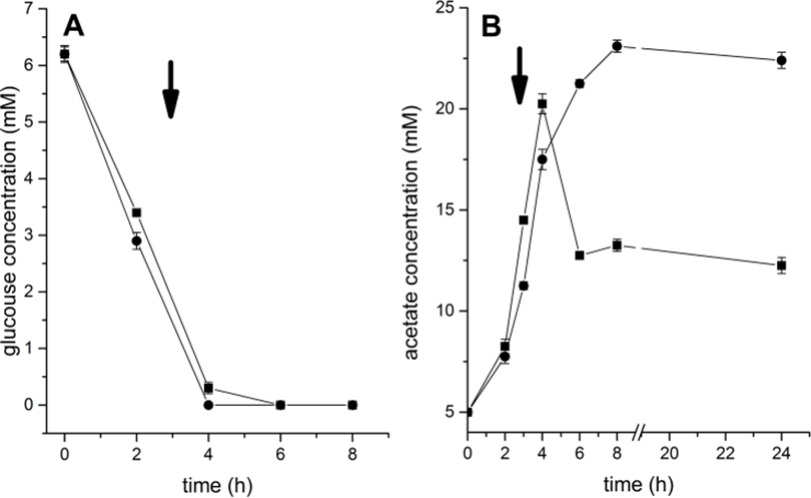
Glucose and acetate contents in the supernatants of growing *S*. *aureus* COL cultures. A) Consumption of glucose in the presence (●) and absence (■) of kendomycin. B) Accumulation/consumption of acetate in the presence (●) and absence (■) of kendomycin. Arrows indicate the addition of kendomycin. The data presented are the mean ± SD of three independent experiments.

Based on the observation that kendomycin decreased the expression of TCA cycle enzymes, we asked whether addition of the compound also affects acetate catabolism. To address this question we next measured the levels of glucose and acetate during growth of strain COL in BHI medium in absence and presence of kendomycin (1.5 x MIC), respectively. As shown in Fig 5A glucose was completely depleted after 3h of cultivation of COL cells, and thus already absent when kendomycin was added. During the same growth period, COL cells secreted acetate into the medium (Fig 5B). However, one hour after depletion of glucose, control COL cells started to utilize acetate for growth, whereas kendomycin treated cells did not (Fig 5B).

## Discussion

In this study, we investigated the antibacterial mode of action of kendomycin in the MRSA strain COL using a multidisciplinary approach. After confirming the growth inhibition effect of kendomycin on the test strain, we performed proteomic and transcriptomic analyses to further investigate the physiological changes in aerobically growing COL cells after kendomycin treatment. Under aerobic growth conditions and in the presence of sufficient amounts of glucose, *S*. *aureus* cells preferentially catabolize glucose via glycolysis, whereas the TCA cycle is repressed [45]. The utilization of glucose via glycolysis generates pyruvate, which under aerobic growth conditions is catabolized to acetyl-CoA and subsequently to acetyl-phosphate. The latter compound is used for substrate-level phosphorylation, and the by-product acetate is secreted into the medium [46]. Because amino acids and glucose are readily available in BHI medium during the early growth stages, the TCA cycle is likely not required (or might be required only to a limited extent) for energy-producing or anabolic processes while a sufficient amount of glucose remains available in the culture medium. However, when glucose becomes depleted from the medium, acetate and amino acids are taken up and used as alternative carbon sources for metabolism primarily via the TCA cycle, which generates the reducing potential for oxidative phosphorylation and biosynthetic intermediate production [46]. To assimilate acetate or amino acids, cells require gluconeogenesis for growth [46,47].In our proteomic and transcriptomic assays, COL cells were challenged with kendomycin during the mid-exponential growth phase (OD_600_ of 2.5–3.5), a time in which acetate continued to accumulate in the medium (Fig 5B), suggesting that the growing *S*. *aureus* cell population still utilized glycolysis as the primary metabolic pathway at this time-point. *S*. *aureus* contains two glyceraldehyde-3-phosphate dehydrogenase (GAPDH) homologs, GapA (syn. GapA1) and GapB (syn. GapA2). GapA is essential for glycolysis, whereas GapB is essential for gluconeogenesis [48]. GapB (GAPDH 2) was down-regulated after kendomycin treatment, as shown by our proteomic analysis, which might explain the prolonged accumulation of acetate in the supernatant of the COL culture after kendomycin treatment in comparison to the control (Fig 5B). In addition, SdhA, one of the subunits of the *S*. *aureus* succinate dehydrogenase enzyme complex, which is the only TCA cycle enzyme that can directly feed electrons into the respiratory chain [29], and PckA, a main gluconeogenic factor, which converts the TCA cycle intermediate oxaloacetate to phosphoenolpyruvate, were found to be down-regulated in kendomycin-treated cells. These findings suggest the breakdown of the TCA cycle and gluconeogenesis in response to the antibiotic challenge. Furthermore, the radioactive labelling experiments indicated a general inhibition of the macromolecule synthesis pathways. The breakdown of the primary metabolic pathway in these cells may lead to the depletion of the energy supply and, consequently, the inhibition of the biosynthetic pathways, which might ultimately lead to growth inhibition and cell death.

Programmed cell death (PCD) is (traditionally) associated with multicellular eukaryotic organisms. However, PCD systems have also recently been reported in bacteria [49,50]. Studies of the *S*. *aureus* LytSR two-component regulatory system led to the identification of the *cid* and *lrg* operons, which affect murein hydrolase activity, stationary phase survival, antibiotic tolerance, biofilm formation, and programmed cell death [51]. In contrast to the *lrg* operon, which inhibits murein hydrolase activity and antibiotic-induced killing, the *cid* operon enhances these processes [51]. The CidA and LrgA proteins are structurally similar to members of the bacteriophage-encoded holin/antiholin system, which comprises a family of small proteins that regulate the timing and onset of phage-induced cell lysis [52,53], and CidA and LrgA have been suggested to exert holin- and antiholin-like functions [13].The putative function of the *cid* operon as an effector of murein hydrolase activity was demonstrated by generating a *cidA* mutant and showing that this strain exhibited decreased murein hydrolase activity [54]. Furthermore, this mutation was also shown to confer tolerance to a variety of antibiotics, including penicillin, rifampin, and vancomycin [54,55]. In the present study, *cidA* expression was clearly up-regulated in the kendomycin-treated cells. In contrast, we detected down-regulated transcription of *lrgA*, which is typically responsible for the negative regulation of hydrolase activity in *S*. *aureus*. However, because we did not detect a significant increase in bacterial cell lysis upon kendomycin treatment, it can be assumed that this compound affects the viability of *S*. *aureus* primarily via holin formation rather than via the modulation of murein hydrolase activity.

In contrast, Clp proteins and other chaperones were the proteins most strongly up-regulated after kendomycin treatment. The importance of differential protein turnover for cell survival has been demonstrated previously [26]. Cells typically use these chaperones and proteases for survival as a compensatory response to stress conditions (especially those that compromise the functionality of proteins). Under stress conditions, the Clp proteins and chaperones such as DnaK, GroEL, ClpB, and ClpC are capable of refolding denatured proteins. Clp proteases are a unique serine protease family, and their sequences are highly conserved between different species [56]. However, the physiological relevance and functions of Clp proteases differ between Gram-negative and Gram-positive bacteria [57]. For example, the deletion of *clpP* does not cause any growth disadvantage in *E*. *coli*. However, the deletion of the same gene in *B*. *subtilis* exerts many effects on cell growth and function, such as reduced growth rate and stress tolerance, filamentous cell morphology, and suppressed competence development [58]. Furthermore, during the last decade, ClpP was reported to play a vital role in the survival and virulence of pathogenic bacteria (including *S*. *aureus*) during host infection [26,59,60], which rendered ClpP as an attractive target for anti-invective compounds and led to the discovery of acyldepsipeptides (ADEPs). ADEPs are a new class of antibiotics that target ClpP activity [61]. It was demonstrated that ADEPs activate ClpP to degrade FtsZ, thereby inhibiting cell division [62]. Although kendomycin appears to inhibit the cell division machinery of the COL strain of *S*. *aureus* as well, its mode of action appears to be markedly different from that of ADEPs. Kendomycin did not induce the swelling of *S*. *aureus* cells (data not shown), as has been reported for ADEPs [62]. Furthermore, our feeding experiments demonstrated that kendomycin completely inhibited DNA, RNA, protein, and cell wall synthesis, in contrast to ADEPs, which have been reported to exert no effect on biopolymer synthesis in bacteria, even at high concentrations (4 x MIC) [62].

Electron microscopic examinations of kendomycin-treated *S*. *aureus* COL cells revealed alterations in the septation process, indicating direct or indirect inhibition of one or more stages of cell division. In bacteria, the formation of a divided septum is a multistep process that is typically initiated by the assembly of the FtsZ protein, a homolog of the eukaryotic protein tubulin, into a ring-shaped structure, the Z-ring, at the midpoint of the cell [63]. The Z-ring is anchored to the cell membrane by several accessory proteins, such as FtsA and ZipA [64]. Because our proteomic approach did not identify an impact of kendomycin on cell division-related proteins, we evaluated the effects of this compound on the transcriptional levels of the *ftsZ* and *ftsA* genes. The transcriptional levels of both genes were down-regulated in kendomycin-treated cells (Fig 3B). It was previously demonstrated that the inactivation of *clpP* in *B*. *subtilis* induced the accumulation of MurAA, an UDP-N-acetylglucosamine 1-caboxyphenyltransferase that catalyzes the first step of the pathway of peptidoglycan synthesis. Gram-negative bacteria only contain a *murA* gene, whereas Gram-positive bacteria contain two separate corresponding genes, *murAA* and *murAB* [65]. Kock et al. showed that in *B*. *subtilis*, MurAA was specifically degraded by the ClpCP protease complex, and this Clp complex-dependent degradation was especially enhanced upon entry into the stationary phase, which caused intermediate growth arrest due to the stalling of murein biosynthesis [58]. In addition, the up-regulation of ClpP not only enhanced the degradation of MurAA in *B*. *subtilis*but also reduced the transcription of the corresponding gene [58]. In line with this report, the transcription of *murAA* was clearly decreased after kendomycin treatment in our study. The kendomycin-mediated down-regulation of 3 genes (*ftsZ*, *ftsA*, and *murAA*) that are known to affect cell division and septum formation in bacteria suggested a direct or indirect effect of the antibiotic on the cell division machinery of *S*. *aureus*. However, additional studies are needed to obtain deeper insight into the mode of action of kendomycin.

## Materials and Methods

### Bacterial strains and growth conditions

*S*. *aureus* COL [66] cells were routinely grown in BHI medium (Difco) or on Mueller-Hinton agar (containing 1.5% agar). All of the bacterial cultures were inoculated from an overnight culture and were diluted to an OD at 600 nm (OD_600_) of 0.1 using fresh BHI medium prior to incubation. To generate aerobic growth conditions, the cells were incubated in Erlenmeyer flasks at a flask-to-medium volume ratio of 10:1 at 37°Cwith shaking at 230 rpm. Aliquots were removed at the indicated time points, and bacterial growth was assessed by measuring the OD_600_.

### Time-killing assay

Single colonies of the COL strain were used to inoculate 100 mL of BHI medium, and the cultures were incubated at 37°C with shaking at 230 rpm for up to 24 h. Kendomycin was added at the indicated time points, and aliquots (200 μL) were removed at various intervals to determine the OD_600_ values. Control samples were treated with an equal amount of methanol, the kendomycin solvent, to control for any side effects of the solvent on growth or cell viability. The CFU/mL of each culture was determined by seeding serial dilutions of aliquots from the culture on Mueller-Hinton agar plates and incubating the plates at 37°C for 24 h.

### Incorporation of radioactive metabolites

An overnight culture grown in casein-yeast-glucose broth (CYG) containing 1 mM of the appropriate unlabeled metabolite was diluted 50-fold using fresh medium and was cultured at 37°C to an OD_600_ of approximately 0.5. The cultures were then divided in half, diluted to an OD_600_ of 0.04 and allowed to regrow to an OD_600_ of 0.1. Subsequently, the appropriate labeled precursor was added to each culture (final concentration of 1μCi/mL), and kendomycin was added (5×MIC) to one of the two cultures. The other culture served as a control after the addition of an equal amount of methanol. The incorporation of the labeled precursor was monitored for up to 1h. Samples of 200 μl were mixed with 2 mL of ice-cold 10% TCA containing 1mM of an unlabeled precursor and were incubated on ice for at least 30 min prior to filtration using glass microfiber filters (Whatman). The filters were washed with 5 mL of TCA (2.5%) containing 50mM of an unlabeled metabolite, dried and counted.

### Protein extraction from whole cell extracts

Treated and untreated cells were harvested by centrifugation one hour after the antibacterial challenge. The cell pellets were washed three times with ice-cold PBS. After the removal of the PBS, the cell pellets were treated with a buffer (2 mL/cell pellet) containing 7 M urea, 2 M thiourea, 4% CHAPS, and 2% w/v DDT. The cells were then incubated on ice for 10 min and were disrupted using a French press. The cell lysates were centrifuged (14,000 rpm for 15 min at 4°C), and the supernatants were placed in new 15-mL Falcon tubes. Protein precipitation was performed using a mixture of acetone and methanol (9:1) chilled to -20°C. The mixture was added (10:1 ratio v/v) drop-wise to the protein solution under shaking. The protein precipitation was completed by incubating the samples overnight at -20°C. The precipitated proteins were collected by centrifugation (4000 rpm for 30 min at 4°C) and washed with 5% H_2_O in acetone. The pellets were then air-dried and dissolved in a minimal amount of DIGE-labeling buffer (approximately 300 μL) containing 7 M urea, 2 M thiourea, 4% CHAPS, and 30 mM Tris-HCl, pH 8.5. After the proteins were completely dissolved, the pH of each sample was tested again using pH strips and (if necessary) was readjusted to pH 8.5 using 1–3 μL of DIGE-labeling buffer, pH 9.5. The protein samples were centrifuged (20,000 rpm for 30 min at 4°C), the supernatants were removed, and the protein concentrations were determined using the Bradford assay. The protein concentrations of all of the samples were then adjusted to 5 mg/mL using DIGE-labeling buffer, pH 8.5. Fifty micrograms of protein from each sample was minimally labeled with one of the different Cy dyes according to the experimental design provided in Table 1.

### 2D analysis of the cellular proteome

As described previously [16], the 2D proteomic analysis, the proteins were loaded into 18-cm pH 4–7 immobilized pH gradient (IPG) strips via overnight passive rehydration. An IPGphor focusing apparatus (Amersham Biosciences) was used for separation using the following program: 50 V step for 3 h, 500 V gradient for 2.5 kVh, 500 V step for 1 kVh, 3500 V gradient for 12 kVh, 3500 V step for 3.5 kVh, 8000 V gradient for 12 kVh, and 8000 V step for 32 kVh. After isoelectric focusing, the IPG strips were equilibrated for 15 min in equilibration buffer (2% SDS, 50 mMTris-HCl [pH 8.8], 6 M urea, 3% glycerol, 0.002% bromophenol blue, 10 mg/mL DTT) for 15 min. The IPG strips were equilibrated again for 15 min in the same buffer, except that the DTT was replaced with iodoacetamide (25 mg/mL). The equilibrated IPG strips were then transferred to 12.5% homogeneous SDS-polyacrylamide gels for 5 h. The gel electrophoresis was performed using an Ettan-DALTsix at 2 W/gel for 45 min and then for 17 W/gel for 4.5 hours. After SDS-PAGE, the gels were scanned at a resolution of 100 μm using a Typhoon 9410 imager (Amersham). The excitation/emission wavelengths for Cy2, Cy3, and Cy5 were 488/520, 532/580, and 633/670 nm, respectively. The 2D gel containing 500 μg of unlabeled pooled standard sample was stained with colloidal Coomassie blue and was scanned using the same imager at an excitation wavelength of 633 nm. Relative protein quantification across all of the samples was performed using DeCyder differential in-gel analysis (DIA) and biological variance analysis (BVA). Student’s *t-*test and a one-way analysis of variance (ANOVA) were performed to identify significant differences in the relative abundances of the protein spots between the samples.

### Peptide mass fingerprinting and MALDI analysis

As described previously [38], differentially expressed protein spots exhibiting a greater than 1.5-fold difference in abundance and a *P* value ≤ 0.01 were excised directly from the gels either using an automated Ettan spot picker (Amersham) or manually. After trypsin in-gel digestion, the tryptic digest was mixed 1:1 with matrix solution (5 mg/mL α-cyano-4-hydroxy-cinnamic acid in 50% acetonitrile and 0.1% TFA), spotted on stainless steel MALDI sample plates and analyzed via MALDI-MS or MALDI-MS-MS using an Applied Biosystems instrument. A database search was performed using Mascot. The protein identification was assigned to the protein spots that displayed a protein score > 70, which correlated with a confidence interval of 99%.

### Real-time reverse transcription-PCR (RT-PCR)

As described in detail previously [67], *S*. *aureus* was grown in BHI medium to the mid-exponential growth phase (OD_600_ of 2.5–3) before kendomycin was added. One hour after the antibacterial challenge, aliquots were removed, and the cells were collected by centrifugation. The bacteria were mechanically disrupted (Fast Prep FP120 instrument; Qbiogene, Heidelberg, Germany), and RNA was isolated (RNeasy Mini Kit; QIAGEN, Hilden, Germany). After treatment with RNase-free DNase I (QIAGEN), the total RNA samples were amplified in an ABI PRISM 7000 Sequence Detection System using SYBR Green PCR master mix (Applied Biosystems, Weiterstadt, Germany) and *gyrB* primers to confirm the absence of gDNA. Previously transcribed cDNA served as a positive control. The RNA samples were then reverse-transcribed (High Capacity cDNA Archive Kit; Applied Biosystems). The same amount of total RNA (2 μg per 25 μL of reaction) from each strain was used to synthesize cDNA, which was then subjected to real-time amplification using specific primers (Table 3) and 62.5 ng of cDNA per 25 μL of reaction. The mRNA expression levels of the different genes were normalized to that of the constitutively expressed internal control *gyrB* [68]. The amount of each transcript was expressed as the *n*-fold difference relative to that of the control gene (2^–DetaC^_T_, where DeltaC_T_ represents the difference in the threshold cycle between the target and control genes).

**Table 3:** Nucleotide sequences of forward and reverse primers that have been used for real time PCR.

No.	Gene	Nucleotide sequence of Forward primer	Nucleotide sequence of reverse primer
1	***clpB***	5’-AGTAGCAGTTAGTGAGCCTGATG-3’	5’-TCTATCTTGAATACGCACACCATG-3’
2	***clpC***	5’-GAAGAAGCAATTCGTTTAAATCATTCA-3’	5’-CTTTCTAATACTTTTGCAGCAATTCCTT-3’
3	***clpP***	5’-TGACAACGTAGCAAATTCAATCGTAT-3’	5’-CACTTCCACCTGGTGAATTAATGTAT-3’
4	***ahpC***	5’-CGTAGTATGCTTCTATCCTGCTGACTT-3’	5’-CATTTACGCCTAATTTTTGTAATTCTT-3’
5	***katA***	5’-GCTGCTGAAATTATAGCTACAGAT -3’	5’-TACTTGAATATACATTGTCCATTT -3’
6	***cidA***	5’-TCGCAGTCATTATCATAGGAACATGT-3’	5’-AAGCGTCTACACCTTTACGATGTTTAT-3’
7	***lrgA***	5’-ATAACATTGCGTTACTCTTCGTACCA -3’	5’-TTGTTGAGACGATTATTAGTCCAATGA-3’
8	***cap5A***	5’-TAGATGAGGTGTCAAAGGACTTAAATGATA-3’	5’-AGTTGCGTATTTTCTTGGTTTGTAATT-3’
9	***cap5C***	5’-TAACATCACATCACTTACATCCTCGATA-3’	5’-GTGCTTGTACTTCCTCTAAGCTTTCA-3’
10	***murAA***	5’-CATCTTTATTAGCTTCTGATAAACCGAGT-3’	5’-TGTATGTAACGTCAGCATTTAAAGTTGTT-3’
11	***ftsZ***	5’- CCACGGAATGAATAATGTTGAATTT -3’	5’- GTGTTAATTTTTCACCGATTTGGAT -3’
12	***ftsA***	5’- GGATACAGAAATCAACGGTTCACATAT -3’	5’- TATAAAACGAATCGGGAACACATTAAT -3’
13	***asp23***	5’-CAAGAACAAAATCAAGAGCCTCAAT-3’	5’-CTTCACGTGCAGCGATACCA-3’

### Measurement of glucose and metabolites in the culture supernatants

These assays were performed as previously described [57]. Briefly, aliquots of bacteria (2 mL) were centrifuged for 5 min at 21,000 x *g* at 4°C at the indicated time points. The culture supernatants were removed and adjusted to pH 8 using KOH. The glucose and acetate concentrations were determined using commercial kits (R-Biopharm AG, Darmstadt, Germany).

### Electron microscopy

Electron microscopy was performed as previously described [69].
